# Network meetings – access to specialist knowledge and mediation of social support for patients with primary brain tumours and their families – a participatory action research project

**DOI:** 10.1186/1472-6955-14-S1-S4

**Published:** 2015-10-08

**Authors:** Pia Riis Olsen

**Affiliations:** 1 Department of Oncology, Aarhus University Hospital, Aarhus, Denmark

## Background

A malignant brain tumour often causes cognitive impairment. This affects both patients and families. Compared to other cancer patients, studies show that they are significantly more in need of social support and help for everyday activities.

A network focused approach has been shown to facilitate the involvement of a supportive social network around the patient and the family, which can assist them in keeping their world together.

## Aims

To develop and implement a research based service that will promote mobilization of a supportive social network for patients with primary brain tumours and their families.

## Methods

Participatory action research involved parallel processes of focus group interview, individual interviews with patients and usually their spouses, and group sessions of co-operative inquiry, education and interaction between the researcher and a group of ten clinical nurses. This group of clinical nurses met with the researcher every quarter to reflect on findings, experiences and practical issues in offering patients the service of personal network meetings.

## Results

The study is estimated to finish in 2015. All interviews are completed and are currently being analysed. The group of nurses has been trained in planning and leading network meetings. At present the service is being piloted in the department and the group of nurses acts as implementation agents in their clinics. Early presentation of individualised network meetings is welcomed as an opportunity and accepted by some patients and relatives. The service is feasible in practice.

## Conclusion

The interactive approach in action research has supported the implementation of the complex service: network meetings. This service has potential in other nursing areas.

